# Quinidine Therapy for Lennox-Gastaut Syndrome With *KCNT1* Mutation. A Case Report and Literature Review

**DOI:** 10.3389/fneur.2019.00064

**Published:** 2019-02-05

**Authors:** Yu Jia, Yicong Lin, Jing Li, Mingyu Li, Yifan Zhang, Yue Hou, Aihua Liu, Liping Zhang, Liping Li, Peng Xiang, Jing Ye, Zhaoyang Huang, Yuping Wang

**Affiliations:** ^1^Department of Neurology, Xuanwu Hospital, Capital Medical University, Beijing, China; ^2^Beijing Key Laboratory of Neuromodulation, Beijing, China; ^3^Department of Pediatrics, Xuanwu Hospital, Capital Medical University, Beijing, China

**Keywords:** quinidine therapy, Lennox-Gastaut syndrome, *KCNT1* mutation, sodium-activated potassium channel, epileptiform discharges

## Abstract

Mutations in the Potassium channel subfamily T member 1 (*KCNT1*) gene have been reported in a range of epileptic encephalopathies. Here we report the case of a 12-year-old male suffering from multiple types of epileptic seizures and cognitive decline from the age of 10. The patient had four types of epileptic seizures, including tonic seizures, atypical absence seizures, myoclonic seizures, and generalized tonic-clonic seizures. The electroencephalogram showed generalized slow spike-and-slow-waves, mutiple-spike-and-slow-waves, as well as short-term fast rhythms bursts. Thus, he was diagnosed with Lennox-Gastaut syndrome. The patient had failed to control seizures after using five first-line antiepileptic drugs. Whole exome sequencing revealed a missense *KCNT1* mutation (c.625 C>T). Previous studies revealed that quinidine could block the *KCNT1* channel. Therefore, we assumed that quinidine might be effective for him. Add-on treatment with quinidine was started when the patient was 12 years old. After an 8-month treatment, the frequency of seizures and epileptiform discharges were significantly reduced. In conclusion, quinidine therapy may offer a new choice for the treatment of Lennox-Gastaut syndrome with *KCNT1* mutations.

## Introduction

Potassium channel subfamily T member 1 *(KCNT1*), also known as *Slack*, is a member of the Slo-type sub family of potassium channel genes (1–3). It has been reported that *KCNT1* mutations were detected in many early onset epileptic encephalopathies, such as epilepsy of infancy with migrating focal seizures (EIMFS) and autosomal dominant nocturnal frontal lobe epilepsy (ADNFLE) (4–7). Previous studies demonstrated that *KCNT1* channels are expressed in neurons and interneurons in the cortex and the CA3 region of the hippocampus (8, 9). Electrophysiological studies have revealed that *KCNT1* mutations enhance the channel-mediated potassium conductance and increase the K^+^ currents in neurons and interneurons, which result in the imbalance between neuronal excitation and inhibition (10, 11). *In vitro* functional studies have shown that these mechanisms may be responsible for epileptogenesis associated with *KCNT1* mutations (10).

Quinidine has been used as a class I antiarrhythmic drug to prevent ventricular arrhythmias. Recent works revealed that quinidine could block the *KCNT1* channel (10, 12). Thus, quinidine is expected to be effective in improving electrophysiological abnormalities caused by *KCNT1* mutations. Recently, there have been several reports about the quinidine treatment of *KCNT1*-related epileptic encephalopathies, such as EIMFS, ADNFLE, and West syndrome (10, 13–16). Quinidine has become a new method for the treatment of *KCNT1*-related epilepsy syndromes.

Here we report a patient suffering from Lennox-Gastaut syndrome with a missense mutation in *KCNT1* (c.625C>T) treated with quinidine. We describe the improvement of the clinical symptoms, the adverse effects, and the dosage adjustment of quinidine during the treatment. Then we review the literature on quinidine treatment of the epilepsy syndrome with *KCNT1* mutation.

## Case Presentation

The patient was a 12-year-old male who had his first seizure attack at the age of 10, and had four types of epileptic seizures. The first type of seizure was tonic axial seizures characterized by flexion of the neck and body and the extension of four extremities for several seconds. The tonic seizures could last several seconds. This type of seizure occurred predominantly at night and the frequency was about 4–5 times per week. The second type was atypical absence seizures, which manifested as a sudden loss of consciousness and the resuming of normal activity right after the seizure. This type of seizure could last about 15 s and the frequency was 4–5 times per week. The third type was the myoclonic seizure, presented as prominent myoclonic jerks of bilateral upper limbs. The frequency of this type of seizures was 2–3 times per day. The last type of seizure was the generalized tonic-clonic seizure, which could last about 5 min. The mean frequency of this type of seizure was <1 time per week.

**Figure 1:**
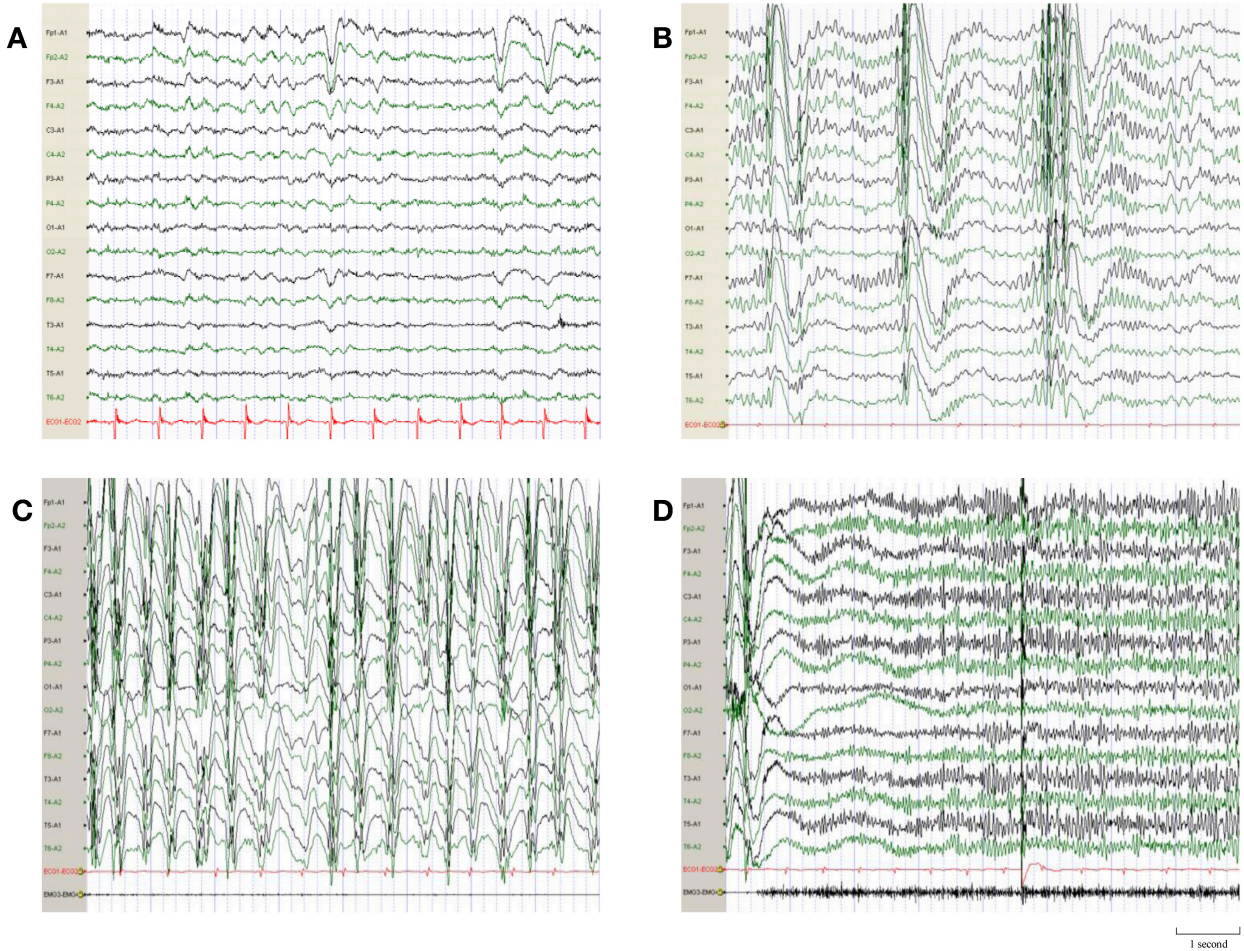
EEG demonstrations before quinidine therapy. **(A)** Interictal EEG before quinidine therapy showed a large number of 3–5 Hz slow waves with middle and high amplitude in the anterior region in background. **(B)** Multiple-spike-and-slow-waves of 0.5–1 Hz were observed during the sleep period. **(C)** Slow spike-and-slow-waves of 1.5–2.5 Hz were observed when the patient was awake. **(D)** Ictal EEG of the tonic seizure showed short-term fast rhythms burst of 16–20 Hz.

The patient was a full-term infant with no history of perinatal asphyxia, head injury, encephalitis, and febrile convulsions. His family history was unremarkable. He had a mild degree of intellectual impairment and learning disability after the onset of the disease. The physical examinations were normal. Auxiliary examinations, including blood routine examination, serum biochemical examination, thyroid function, autoimmunity antibody, and blood ammonia, were normal. The electrocardiogram was normal and the QT interval (QTc) was 372 ms. There was no lesion on the brain MRI. The Wechsler Intelligence Scale showed a borderline cognition impairment. The electroencephalogram (EEG) before treatment showed that there was a large number of 3 to 5 Hz slow waves with middle and high amplitude in the anterior region in the background. Fast rhythms bursts of 16 to 20 Hz and multiple-spike-and-slow-waves of 0.5 to 1 Hz were observed during the sleep period. Slow spike-and-slow-waves of 1.5 to 2.5 Hz were observed during awake time (Figure 1).

The patient was diagnosed with Lennox-Gastaut syndrome after considering his multiple types of epileptic seizures, mental retardation, and typical electroencephalographic features. He was refractory to a multiple anti-epileptic drugs treatment, including sodium valproate (8 mg/kg/day), levetiracetam (50 mg/kg/day), clonazepam (0.0375 mg/kg/day), topiramate (3.75 mg/kg/day), and lamotrigine (2.5 mg/kg/day). Whole exome sequencing (WES) identified a novel heterozygous *KCNT1* mutation (chr9:138649026; c.625C>T; p.Arg209Cys) inherited from his father. This missense mutation was highly likely to cause the dysfunction of the *KCNT1* channel and led to a gain-of-function phenotype. This alteration had not been previously reported and was not found in the ExAC database (http://exac.broadinstitute.org/), and was predicted to be likely pathogenic.

This study was approved by the human research ethic committees of Xuanwu hospital capital medical university. Written informed consent was obtained from all participants and guardians of minors for the quinidine therapy and the publication of this study. Additive quinidine therapy to our patient was initiated at 12 years of age. The doses of the above anticonvulsants remained unchanged. In the month before quinidine therapy, the patient had 16 tonic seizures, 12 atypical absence seizures, 10 myoclonic seizures, and 1 generalized tonic-clonic seizures.

**Figure 2:**
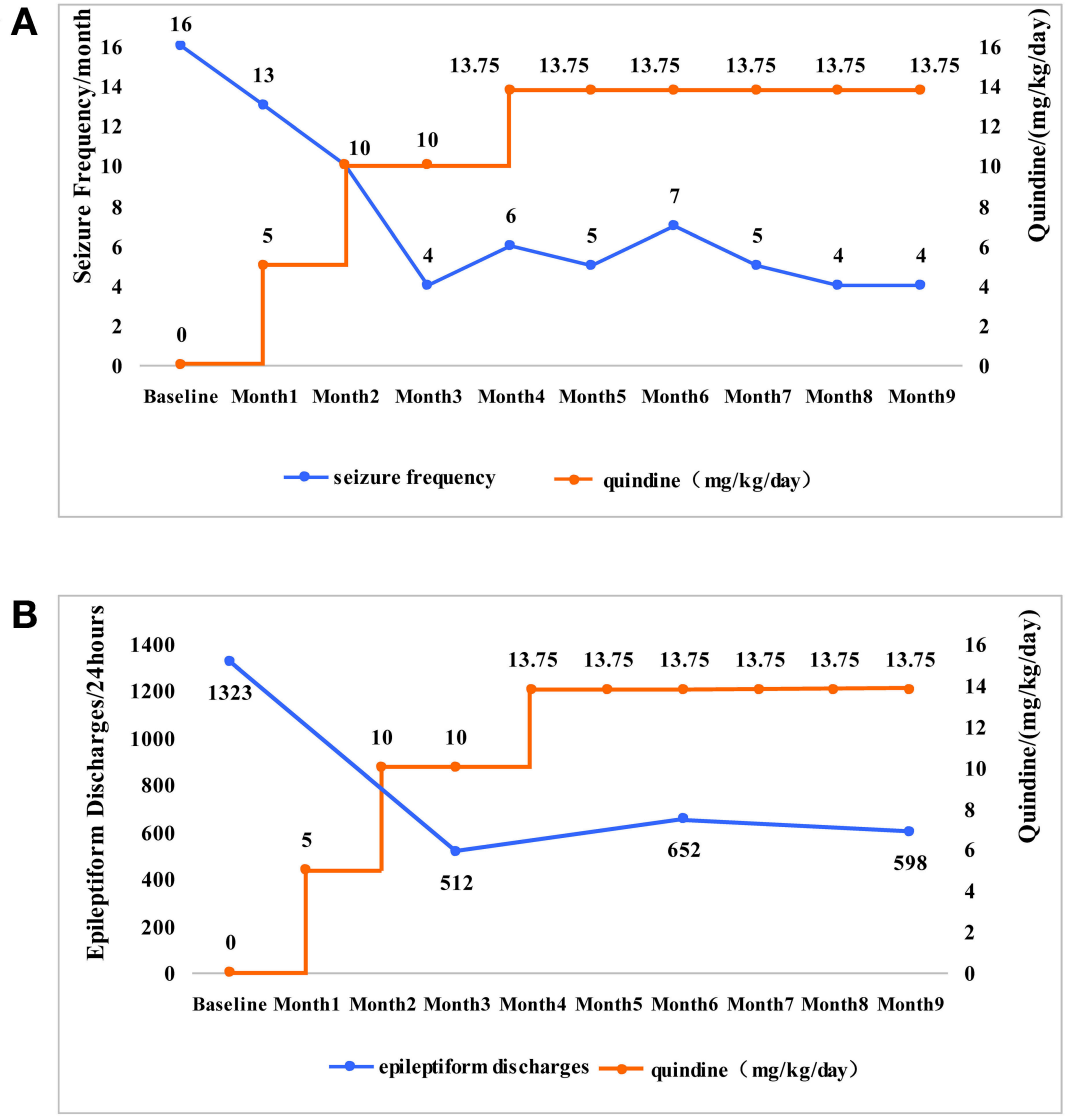
The changes of the seizure frequency and epileptiform discharges along with the changes of quinidine dosages. **(A)** The patient was given quinidine at a starting dosage of 5 mg/kg/day. The dose slowly increased and maintained at 13.75 mg/kg/day. As the dosage of quinidine increased, the frequency of tonic seizures (the main type of seizures in the patient) gradually reduced from 16 times per month to 4 times per month. **(B)** The epileptiform discharge of EEG was 1,323 times per 24 h before taking the quinidine. The epileptiform discharge was 512 times per 24 h in the 3rd month, 652 times per 24 h in the 6th month, 598 times per 24 h in the 9th month. With the increase of dosages, the epileptiform discharge showed a significant reduction.

After admission, the quinidine therapy was initiated with 5 mg/kg/day in 3 divided doses under electrocardiographic (ECG) monitoring. The QTc ranged from 361 to 415 ms, with an average of 378 ms (the normal limit of QTc is within 450 ms). After 1 month of treatment, the dose of quinidine was titrated to 10 mg/kg/day and he had 13 tonic seizures during this month. The dose was maintained during the following 2 months. QTc was in normal range (391–436 ms). There was also no other adverse effect of quinidine. At the fourth month, the dose of quinidine was increased to 13.75 mg/kg/day in 3 divided doses. The frequency of tonic seizures ranged between 4 and 6 times per month. The mean QTc interval was 383 ms. As no adverse effects were experienced, the dose of quinidine was maintained to 13.75 mg/kg/day during the following 4 months. The patient had 4 tonic seizures per month. The frequency of tonic seizures subsided by 75% (Figure 2A), whereas the frequency of the other types of seizures was not reduced significantly.

Video EEG (VEEG) was performed regularly during treatment and the number of epileptic discharges was counted by four expert technicians who had not seen the patient's clinical information. One slow spike-and-wave complex or one episode of paroxysmal fast rhythms in EEGs were counted as one epileptiform discharge. 1323 epileptiform discharges were recorded during a 24-h video EEG before the treatment of quinidine. We subsequently applied the 24-h VEEG every 3 months to evaluated the efficacy of the quinidine treatment. The total number of epileptic discharges was 512, 652, and 598 in the next 3, 6, and 9 months, respectively. The epileptiform discharges decreased by 54.80% (Figure 2B).

## Discussion

In this case, the patient had four types of epileptic seizures, including tonic seizures, atypical absence seizures, myoclonic seizures, and generalized tonic-clonic seizures. The EEG before treatment showed generalized slow spike-and-slow-waves, multiple-spikes-and-slow-waves, and short-term fast rhythms bursts. He was diagnosed with Lennox-Gastaut syndrome. The WES identified a missense *KCNT1* mutation (c.625 C>T). Quinidine was reported to be effective in patients with KCNT1 mutations. Thus, we applied this drug to our patient. After a treatment period of 8 months, the frequency of tonic seizures and epileptiform discharges decreased significantly.

Up to now, the exact mechanism of *KCNT1*-related epilepsy is still unknown. Previous studies suggested that this mutation resulted in a gain of function in this potassium channel, and the magnitude of gain of function correlated with the clinical severity (10). It has been confirmed that quinidine can block *KCNT1* channels *in vitro* (10, 12, 17, 18). In our study, the patient was treated with quinidine for 8 months and the frequency of seizures and epileptiform discharges were significantly reduced. Thus, quinidine therapy was effective for our patient. Our study suggested that quinidine therapy might offer a new method for the treatment of *KCNT1*-related epilepsy syndromes.

We summarized a total number of 16 unrelated cases [15 sporadic patients and 5 patients of a pedigree (13–16, 19–24)] with *KCNT1*-related epilepsy syndrome treated with the quinidine reported previously (Table 1). The mutation sites were c.1887G>C; p.K629N, c.1283G>A; p.R428Q (recurrent in two unrelated patients), c.808C>C/G; p.Q270E, c.2965G>T, c.2849G>A; p.R950Q, c.1955G>T; p.G652V, p.R428Q, c.1421G>A, c.2386T>C; p.Y796H, c.1193G>A, c.820C>A, p.L274I, c.2849G>A; p.R950Q, c.2677G>A; p.E893K and c.2782C>T; p.R928C. All *KCNT1* mutations reported were heterozygous missense mutations except for those not mentioned in the literature. In these 16 cases, the treatments were effective in 8 cases (13–16, 19) and the others were ineffective (Effective response was defined as >50% reduction in seizure frequency) (14, 20–24).

**Table 1:** Summary of quinidine in the treatment of *KCNT1* gene mutation related-epilepsy.

**No**.	**Author & year**	**Seizure type (syndrome)**	**Sex**	**Age of onset**	**Mutation site**	**Heterozygous mutation**	**Age for quinidine therapy**	**Quinidine starting dosage**	**Quinidine maintenance dosage**	**Prolonged QT_**C**_ interval**	**Other side effects**	**Therapeutic effects**
Patient 1	Mikati et al. (14)	Focal seizures and status epilepticus (EIMFS)	Male	4 months old	*c.1887G>C; p.K629N*	Not mentioned	3 years old	12 mg/kg/day	34.4 mg/kg/day	Not mentioned	No	Seizure frequency decreased by 80%.
Patient 2	Bearden et al. (15)	Focal seizures (EIMFS)	Female	10 weeks old	*c.1283G>A; p.R428Q*	Yes	25 months old	2 mg/kg/day	42 mg/kg/day	Not mentioned	No	Seizure free
Patient 3	Fukuoka et al. (13)	Epileptic spasms (West Syndrome)	Male	5 months old	*c.1955G>T; p.G652V*	Yes	2 and a half years old	2 mg/kg/day	60 mg/kg/day	No	No	Seizure frequency decreased by 70%.
Patient 4	Dilena et al. (19)	Focal seizures (EIMFS)	Male	2 days	*c.2849G>A; p.R950Q*	Yes	3 and half months old	Not mentioned	45 mg/kg/day	Yes	Not mentioned	Seizure frequency decreased by 90%.
Patient 5	Dilena et al. (19)	Focal seizures (EIMFS)	Male	1 day	*c.2677G>A; p.E893K*	Yes	16 months old	Not mentioned	58 mg/kg/day	No	Not mentioned	Seizure frequency decreased by 90%.
Patient 6	Shang et al. (16)	EIMFS	Not mentioned	Not mentioned	Not mentioned	Not mentioned	Not mentioned	Not mentioned	Not mentioned	Not mentioned	Not mentioned	Seizure frequency decreased.
Patient 7	Shang et al. (16)	EIMFS	Not mentioned	Not mentioned	Not mentioned	Not mentioned	Not mentioned	Not mentioned	Not mentioned	Not mentioned	Not mentioned	Seizure frequency decreased.
Patient 8	Abdelnour et al. (20)	Multiple seizures (EIFMS)	Male	3 days old	*c.2965G>T*	Yes	3 months old	10 mg/kg/day	39 mg/kg/day	Yes	No	Seizure frequency decreased.
Patient 9	Mullen et al. (21)	ADNFLE	Not mentioned	3 years old	*c.2849G>A; p.R950Q*	Yes	28 years old	Not mentioned	Not mentioned	Yes	Not mentioned	Ineffectiveness
Patient 10	Madaan et al. (22)	Focal seizures (EIMFS)	Male	3 days old	*c.808C>C/G; p.Q270E*	Yes	6 months old	10 mg/kg/day	35 mg/kg/day	Yes	No	Ineffectiveness
Patient 11	Chong et al. (23)	Hemiclonicseizures	Male	6 weeks old	*p.R428Q*	Yes	5 years old	34 mg/kg/day	73 mg/kg/day	Not mentioned	Not mentioned	Ineffectiveness
Patient 12	Abdelnour et al. (20)	Asymmetric tonic seizures	Male	Few days afterbirth	*c.1421G>A*	Yes	13 years old	4 mg/kg/day	37.5 mg/kg/day	Yes	No	Ineffectiveness
Patient 13	Mikati et al. (14)	Nocturnal generalized tonic–clonic seizures	Female	1 and a half years old	*c.2386T>C; p.Y796H*	Yes	11 years old	1 mg/kg/day	40 mg/kg/day	Yes	No	Ineffectiveness
Patient 14	Abdelnour et al. (20)	Tonic seizures and generalized tonic–clonic seizures	Male	4 years old	*c.1193G>A*	Yes	9 years old	11 mg/kg/day	60 mg/kg/day	Yes	No	Ineffectiveness
Patient 15	McTague et al. (24)	Focal seizures (EIMFS)	Not mentioned	1 day	*c.820C>A; p.L274I*	Yes	Not mentioned	Not mentioned	40 mg/kg/day	Not mentioned	Not mentioned	Ineffectiveness
Pedigree 1	Mullen et al. (21)	ADNFLE	Not mentioned	From 2 years old to 15 years old	*c.2782C>T; p.R928C*	Yes	From 15 years old to 54 years old	Not mentioned	Not mentioned	Yes	Not mentioned	Ineffectiveness

*EIMFS, epilepsy of infancy with migrating focal seizures; ADNFLE, autosomal dominant nocturnal frontal lobe epilepsy*.

There were 9 previously reported patients who suffered from EIMFS with *KCNT1* mutation and treated by quinidine. The treatment was effective in 7 cases and ineffective in 2 cases. Interestingly, patient 2 and patient 11 had the same *KCNT1* mutation (p.R428Q), but the therapeutic effects were completely opposite. Patient 2 of Bearden et al. (15) was a 3-year-old female patient of EIMFS, who became seizure-free after treatment. Patient 11 of Chong et al. (23) was a 5-year-old male patient who suffered from an unclassified early onset epileptic encephalopathy. In this latter case, the seizure frequency did not decrease significantly. In addition, Mullen et al. (21) studied the effectiveness of quinidine for six patients (Patient 7 and five patients of the pedigree) who suffered from ADNFLE with *KCNT1* mutations. They found that the seizure frequency of these six patients decreased by less than 50%. It was reported that one patient who suffered from West Syndrome with *KCNT1* mutation benefited from quinidine therapy. Thus, compared to ADNFLE and other epileptic syndromes, quinidine therapy tended to be more effective in EIMFS and West syndrome in patients who carried the *KCNT1* mutation. Therefore, we assumed that the epilepsy phenotype was likely associated with the therapeutic effect.

Moreover, in a recent study by Abdelnour et al. (20) we noticed that the response of quinidine therapy may be age-dependent; younger patients may respond better to this therapy (20). In 16 unrelated cases reported, the mean age of 8 cases responded, except for those whose ages were not identified in the literature, was 2.11 ± 1.18 years old (the range was between 3 months and 3-and-a-half years old) (13–16, 19), and the mean age of the other cases not responded was 21.13 ± 15.92 years old (the range was between 6 months and 54 years old) (14, 20–24). Using a cut-off of 4 years of age, 6/7 patients < 4 years of age responded, and 0/11 patients > 4 years responded. These data revealed that efficacy of quinidine for *KCNT1*-related epilepsy might also depend on the age at quinidine therapy initiation. In our case, the patient was diagnosed as suffering from Lennox-Gastaut syndrome, and quinidine treatment was initiated at 12 years of age. The quinidine therapy proved to be significantly effective. The therapeutic effect of quinidine may be influenced by multiple factors. The epilepsy phenotype, initiation age of therapy, and prior neuronal injury, may all play a role in the efficacy of quinidine therapy. Henceforth, randomized controlled trials (RCT) should be performed to identify the relationship between the influencing factors and the efficacy of quinidine therapy.

So far, there is still no consensus on the effective dosage of quinidine for *KCNT1*-related epilepsy. The therapeutic dosage of quinidine in the treatment of pediatric cardiac disease is 15–60 mg/kg/day, and the maximum daily dose is 3,000–4,000 mg (14, 25, 26). In our case, we used the initial amount of 5 mg/kg/day. The dosage was slowly increased to 13.75 mg/kg/day. No adverse effects of quinidine were reported, and the frequency of tonic seizures and epileptiform discharges decreased significantly. Thus, we maintained this dose for treatment. Further studies are needed to determine the suitable dosage of quinidine for the treatment of *KCNT1*-related epilepsy.

Previously, it was believed that the penetration was complete in patients with *KCNT1* mutations. However, it was reported that there was one individual from an ADNFLE family with a *KCNT1* mutation without causing a phenotype (7). The authors proposed that this individual's unaffected status was most likely due to incomplete penetrance or non-penetrance (7). For our patient, the WES identified a missense *KCNT1* mutation. He was inferred to inherit a *KCNT1* mutation from his unaffected father. We assumed that his father's unaffected status might be due to incomplete penetrance or non-penetrance. Because his grandparental samples were unavailable, we could not confirm the origin of the mutations.

## Conclusions

In summary, we used quinidine to treat a patient with *KCNT1*-related Lennox-Gastaut syndrome, and the seizure frequency and epileptiform discharges were significantly reduced. Quinidine provided valuable clinical experience for the individualized treatment for epilepsy patients with *KCNT1* mutations. Nevertheless, there are too few cases reported with quinidine treatment for *KCNT1*-related epilepsy syndromes. Thus, further studies are required to confirm the effectiveness and the suitable dosage of quinidine therapy for *KCNT1*-related epilepsy syndromes.

## Ethics Statement

All procedures were approved by the ethics committee of Xuanwu Hospital. The parents of our patient provided written informed consent.

## Author Contributions

YJ, ZH, and YL were the major contributors in writing the manuscript. YW, JY, LZ, and AL contributed to the diagnosis and treatment of the patient. LL contributed to the EEG analysis. JL, ML, PX, and YZ contributed to counting the number of epileptic discharges. JY, ZH, and YH contributed to the analysis of genetic examination. JY and ZH contributed to checking the manuscript. All authors read and approved the final manuscript.

### Conflict of Interest Statement

The authors declare that the research was conducted in the absence of any commercial or financial relationships that could be construed as a potential conflict of interest.

## Abbreviations

EIMFS: epilepsy of infancy with migrating focal seizures
ADNFLE: autosomal dominant nocturnal frontal lobe epilepsy
EEG: electroencephalogram
WES: whole exome sequencing
PMA: phorbol-12-myristate-13-acetate.

## References

[1] Bhattacharjee A Kaczmarek LK. For K^+^ channels, Na^+^ is the new Ca^2+^. Trends Neurosci. (2005) 28:422–8. 10.1016/j.tins.2005.06.003 15979166

[2] Yuan A Santi CM Wei A Wang ZW Pollak K Nonet M. The sodium-activated potassium channel is encoded by a member of the Slo gene family. Neuron (2003) 37:765–73. 10.1016/S0896-6273(03)00096-5 12628167

[3] Kim GE Kaczmarek LK. Emerging role of the KCNT1 slack channel in intellectual disability. Front Cell Neurosci. (2014) 8:209. 10.3389/fncel.2014.00209 25120433 PMC4112808

[4] Heron SE Smith KR Bahlo M Nobili L Kahana E Licchetta L. Missense mutations in the sodium-gated potassium channel gene KCNT1 cause severe autosomal dominant nocturnal frontal lobe epilepsy. Nat Genet. (2012) 44:1188–90. 10.1038/ng.2440 23086396

[5] Barcia G Fleming MR Deligniere A Gazula VR Brown MR Langouet M. De novo gain-of-function KCNT1 channel mutations cause malignant migrating partial seizures of infancy. Nat Genet. (2012) 44:1255–9. 10.1038/ng.2441 23086397 PMC3687547

[6] Coppola G. Malignant migrating partial seizures in infancy: an epilepsy syndrome of unknown etiology. Epilepsia (2009) 50(Suppl. 5):49–51. 10.1111/j.1528-1167.2009.02121.x 19469847

[7] Møller RS Heron SE Larsen LH Lim CX Ricos MG Bayly MA. Mutations in KCNT1 cause a spectrum of focal epilepsies. Epilepsia (2015) 56:e114–20. 10.1111/epi.13071 26122718 PMC5915334

[8] Bhattacharjee A Gan L Kaczmarek LK. Localization of the slack potassium channel in the rat central nervous system. J Comp Neurol. (2002) 454:241–54. 10.1002/cne.10439 12442315

[9] Joiner WJ Tang MD Wang LY Dworetzky SI Boissard CG Gan L. Formation of intermediate-conductance calcium-activated potassium channels by interaction of slack and slo subunits. Nat Neurosci. (1998) 1:462–9. 10.1038/2176 10196543

[10] Milligan CJ Li M Gazina EV Heron SE Nair U Trager C. KCNT1 gain of function in 2 epilepsy phenotypes is reversed by quinidine. Ann Neurol. (2014) 75:581–90. 10.1002/ana.24128 24591078 PMC4158617

[11] Kim GE Jack K Giulia B Quraishi IH Martin HC Edward B. Human slack potassium channel mutations increase positive cooperativity between individual channels. Cell Rep. (2014) 9:1661–72. 10.1016/j.celrep.2014.11.015 25482562 PMC4294418

[12] Yang B Gribkoff VK Pan J Damagnez V Dworetzky SI Boissard CG. Pharmacological activation and inhibition of slack (Slo2.2) channels. Neuropharmacology (2006) 51:896–906. 10.1016/j.neuropharm.2006.06.003 16876206

[13] Fukuoka M Kuki I Kawawaki H Okazaki S Kim K Hattori Y Quinidine therapy for west syndrome with KCNT1 mutation: a case report. Brain Dev Jpn. (2016) 39:80–3. 10.1016/j.braindev.2016.08.002 27578169

[14] Mikati MA Jiang YH Carboni M Shashi V Petrovski S Spillmann R. Quinidine in the treatment of KCNT1-positive epilepsies. Ann Neurol. (2016) 78:995–9. 10.1002/ana.24520 26369628 PMC4811613

[15] Bearden D Strong A Ehnot J DiGiovine M Dlugos D Goldberg EM. Targeted treatment of migrating partial seizures of infancy with quinidine. Ann Neurol. (2014) 76:457–61. 10.1002/ana.24229 25042079

[16] Shang K Zhang Y Yang X Liu A Yang Z Liu X. Clinical features and gene mutations in epilepsy of infancy with migrating focal seizures. Zhonghua Er Ke Za Zhi. (2016) 54:735–9. 10.3760/cma.j.issn.0578-1310.2016.10.005 27784474

[17] Santi CM Ferreira G Yang B Gazula VR Butler A Wei A. Opposite regulation of slick and slack K^+^ channels by neuromodulators. J Neurosci. (2006) 26:5059–68. 10.1523/JNEUROSCI.3372-05.2006 16687497 PMC6674240

[18] Bhattacharjee A Joiner WJ Wu M Yang Y Sigworth FJ Kaczmarek LK. Slick (Slo2.1), a rapidly-gating sodium-activated potassium channel inhibited by ATP. J Neurosci. (2003) 23:11681–91. 10.1523/JNEUROSCI.23-37-11681.2003 14684870 PMC6740956

[19] Dilena R DiFrancesco JC Soldovieri MV Giacobbe A Ambrosino P Mosca I. Early treatment with quinidine in 2 patients with epilepsy of infancy with migrating focal seizures (EIMFS) due to gain-of-function KCNT1 mutations: functional studies, clinical responses, and critical issues for personalized therapy. Neurotherapeutics (2018) 15:1112–26. 10.1007/s13311-018-0657-9 30112700 PMC6277296

[20] Abdelnour E Gallentine W McDonald M Sachdev M Jiang Y Mikati MA. Does age affect response to quinidine in patients with KCNT1 mutations? Report of three new cases and review of the literature. Seizure (2018) 55:1–3. 10.1016/j.seizure.2017.11.017 29291456

[21] Mullen SA Carney PW Roten A Ching M Lightfoot PA Churilov L. Precision therapy for epilepsy due to KCNT1 mutations: a randomized trial of oral quinidine. Neurology (2017) 90:e67–2. 10.1212/WNL.0000000000004769 29196578

[22] Madaan P Jauhari P Gupta A Chakrabarty B Gulati S. A quinidine non responsive novel KCNT1 mutation in an Indian infant with epilepsy of infancy with migrating focal seizures. Brain Dev. (2018) 40:229–32. 10.1016/j.braindev.2017.09.008 29037447

[23] Chong PF Nakamura R Saitsu H Matsumoto N Kira R. Ineffective quinidine therapy in early onset epileptic encephalopathy with KCNT1 mutation. Ann Neurol. (2016) 79:502–3. 10.1002/ana.24598 26748457

[24] McTague A Nair U Malhotra S Meyer E Trump N Gazina EV. Clinical and molecular characterization of KCNT1-related severe early-onset epilepsy. Neurology (2018) 90:e55–66. 10.1212/WNL.0000000000004762 29196579 PMC5754647

[25] Cohen IS Jick H Cohen SI. Adverse reactions to quinidine in hospitalized patients: findings based on data from the boston collaborative drug surveillance program. Prog Cardiovasc Dis. (1977) 20:151–63. 10.1016/0033-0620(77)90004-4 331400

[26] Luedtke SA Kuhn RJ Mccaffrey FM Pharmacologic management of supraventricular tachycardias in children. Part 2: atrial flutter, atrial fibrillation, and junctional and atrial ectopic tachycardia. Ann Pharmacother. (1997) 31:1347–59. 10.1177/106002809703101016 9391691

